# Analysis of the Use and Applicability of Different Variables for the Prescription of Relative Intensity in Bench Press Exercise

**DOI:** 10.3390/biology11020336

**Published:** 2022-02-21

**Authors:** José Luis Maté-Muñoz, Manuel Vicente Garnacho-Castaño, Juan Hernández-Lougedo, Luis Maicas-Pérez, Raúl Notario-Alonso, Marzo Edir Da Silva-Grigoletto, Pablo García-Fernández, Juan Ramón Heredia-Elvar

**Affiliations:** 1 Department of Radiology, Rehabilitation and Physiotherapy, Complutense University of Madrid, 28040 Madrid, Spain; jmate03@ucm.es; 2 Sant Joan de Déu Teaching Campus, 08034 Barcelona, Spain; manuelvicente.garnacho@sjd.edu.es; 3 Department of Physical Activity and Sports Science, Alfonso X El Sabio University, 28691 Madrid, Spain; jhernlou@uax.es (J.H.-L.); lmaicper@uax.es (L.M.-P.); rnotario@uax.es (R.N.-A.); jelvaher@uax.es (J.R.H.-E.); 4 Functional Training Group, Post Graduate Program in Physical Education, Department of Physical Education, Federal University of Sergipe, São Cristóvão 49100-000, Brazil; medg@ufs.br

**Keywords:** strength, sport performance, human performance, velocity, load, training, repetitions, fatigue

## Abstract

**Simple Summary:**

The aim of this research is to analyze the different variables that influence the prescription of resistance training (one-repetition maximum (1RM) and number of maximal repetitions (xRM)) through the velocity of execution, with the aim of approaching the precise definition and control of intensity in bench press exercise. Fifty male physical education students were divided into four groups according to their relative strength ratio (RSR) and performed a 1RM bench press test, and two maximum number of repetitions (MNR) tests one week apart, using a relative load corresponding to 70% 1RM determined through the mean propulsive velocity (MPV) obtained from the individual load–velocity relationship. Regarding MPV, the best (fastest) repetition of the set values were similar between groups (0.62 m·s^−1^–0.64 m·s^−1^). The average MNR was 12.38 ± 2.51, with significant variation between groups with regards to MNR (CV:13–29%), and greater variability in the group corresponding to the lowest RSR values (CV: 29%). The use of variables such as the 1RM or a MNR do not allow an adequate degree of precision to prescribe and control the relative intensity of resistance training. Besides, execution velocity control can offer an adequate alternative to guarantee an accurate prescription of intensity with regard to resistance training.

**Abstract:**

**Background**: The aim of the study was to analyze the use of variables such as % of one-repetition maximum (1RM) and number of maximal repetitions (xRM) with execution velocity to define and control the intensity of resistance training in bench press exercise. Hence, exercise professionals will achieve better control of training through a greater understanding of its variables. **Methods:** In this cross-sectional study, fifty male physical education students were divided into four groups according to their relative strength ratio (RSR) and performed a 1RM bench press test (T1). In the second test, participants performed repetitions to exhaustion (T2), using a relative load corresponding to 70% 1RM determined through the mean propulsive velocity (MPV) obtained from the individual load–velocity relationship. This same test was repeated a week later (T3). Tests were monitored according to the MPV of each repetition and blood lactate values (LACT). **Results**: Regarding MPV, the best (fastest) repetition of the set (MPVrep Best) values were similar between groups (0.62 m·s^−1^–0.64 m·s^−1^), with significant differences in relation to the high RSR group (*p* < 0.001). The average maximum number of repetitions (MNR) was 12.38 ± 2.51, with no significant differences between the RSR groups. Nonetheless, significant variation existed between groups with regards to MNR (CV: 13–29%), with greater variability in the group corresponding to the lowest RSR values (CV: 29%). The loss of velocity in the MNR test in the different groups was similar (*p* > 0.05). Average LACT values (5.72 mmol·L^−1^) showed significant differences between the Medium RSR and Very Low RSR groups. No significant differences were found (*p* > 0.05) between T2 and T3 with regards to MNR, MPVrep Best, or MPVrep Last, with little variability seen between participants. **Conclusions**: The use of variables such as the 1RM, estimated using an absolute load value, or an MNR do not allow an adequate degree of precision to prescribe and control the relative intensity of resistance training. Besides, execution velocity control can offer an adequate alternative to guarantee an accurate prescription of intensity with regard to resistance training.

## 1. Introduction

Despite the huge amount of research and literature available on resistance training and achieving the correct stimulus, knowledge about the effort level achieved through any type of training oriented towards improving strength remains an insufficiently addressed issue [1,2,3]. This fact, which limits the methodological quality of the existing literature, possesses implications concerning both the challenge of establishing an appropriate dose–response relationship and the information available to professionals using physical exercise as an intervention (physicians, physiotherapists, coaches). This latter aspect is particularly important in the context of exercise prescription.

Nowadays, the absolute output obtained in a progressive load test until the one-repetition maximum (1RM) is reached or, failing that, a determined number of maximal repetitions (xRM) continue to be used as “gold standard” indicators to define and manage the intensity of resistance training. In these cases, it is assumed that the same % of the 1RM in absolute terms, obtained through a 1RM test [4,5,6], or a similar number of maximum repetitions (xRM) lead to the same relative intensity being reached within the same group of individuals, being considered in the scientific literature as a valid test for the assessment of strength [7,8,9].

On the other hand, in recent years the emergence of variables such as execution velocity has led to important advances in the knowledge of training load monitoring [10,11]. This valuable contribution has not always been entirely understood or broken down with regard to its potential application for coaches. This can be seen through the fact that many coaches continue to use indicators based on the absolute load values discussed previously [12]. In addition, in cases where the connection is made with velocity, the approach applied shares the same limitations as traditional methodologies [13,14]. Such limitations impair both advancement in the understanding of this variable and its use by exercise professionals.

Thus, the applicability of traditional approaches such as 1RM is difficult because they require considerable time and effort to determine [15,16]. Moreover, the adaptation of the individual varies according to the values obtained in the 1RM test, requiring frequent reevaluations, which makes its use difficult. On the other hand, when we use the execution velocity, it may be easier to align the training with the individual’s needs, due to the proximity between the maximum velocity and the percentage of 1RM, expressing the real capacity of the individual. Therefore, absolute loads below the load associated with maximum velocity will be more closely related to the relative intensity prescribed in the training session.

With regard to exercise prescription based on xRM (e.g., 8RM, and 12RM, etc.), it is proposed that it would serve to consider, besides the aforementioned issues, potential variability between individuals at the same relative intensity level [17,18,19,20]. Such variability could invalidate the use of xRM as a means to prescribe the relative intensity of training within a group of individuals, especially when considering the significant differences found in maximum performance capacity. Further, the adaptation reserve of each individual should be taken into account. Analysis of this variability and its relationship with execution velocity would enable knowledge to advance about the use of these variables.

Therefore, we analyze the use of variables such as % of 1RM and xRM with execution velocity to define and control the intensity of strength training. Hence, exercise professionals will achieve better control of training through a greater understanding of its variables. This would not be achievable through methods based on the estimation of the absolute load. Such knowledge would be impossible to achieve via traditional means based on the estimation of absolute load. All of this will lead to more optimal outcomes regarding understanding of the processes related to the definition of training variables used in research, while also improving knowledge application by exercise professionals in their day-to-day contact with athletes and/or patients.

Thus, the aims of the present study were the following: (1) Analyze the velocity values produced in a progressive load test until 1RM in a bench press exercise (BP). (2) Analyze the different velocity values produced in a BP exercise, according to strength level, via a test in which as many repetitions as possible are performed until exhaustion (maximum number of repetitions (MNR)). The MNR test was performed against the 70% 1RM load, which was determined from the individual load–velocity relationship. Thus, a target MPV to be attained in the first repetition (usually the fastest) of the set in each session was used as an estimate of 70% 1RM. (3) Examine intra- and inter-individual variability in the different velocity variables produced from the MNR test to determine the viability of using the xRM value as an indicator of the relative intensity of an upper-limb push exercise.

## 2. Materials and Methods

### 2.1. Study Design

**Figure 1:**
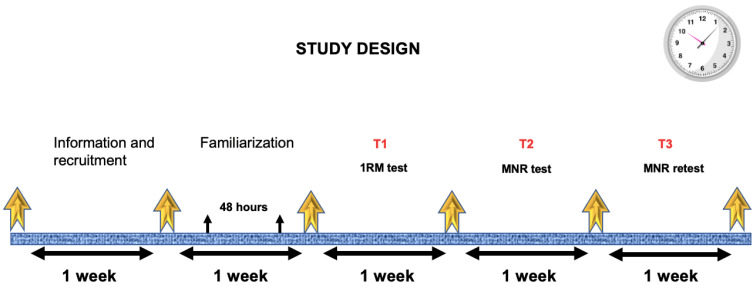
Study design. MNR = maximum numbers of repetitions.

In the present cross-sectional study, participants performed three testing sessions one week apart. The first test was a progressive load test on the bench press (BP) up to 1RM (T1) and served to identify the load–velocity relationship for each participant. In the second test, participants performed repetitions to exhaustion (T2), using relative loads that had been determined in the prior 1RM test. The relative load selected corresponded to 70% 1RM determined through the mean propulsive velocity (MPV) obtained from the individual load–velocity relationship. In this test, each individual performed as many repetitions as possible (MNR) until the point of muscular exhaustion. The third test (T3), performed one week later, required participants to repeat the prior test to exhaustion (T2) to examine intra-individual variability in MNR. Participants who completed the three sessions did so either in the morning or in the afternoon, within the same time frame (±2 h) and in the same environmental conditions (at a temperature of between 18 and 22 °C and at 40–55% humidity). In the previous week, two familiarization sessions were performed to get comfortable with the BP exercise. Each participant performed 4 × 10 repetitions with a 10 kg barbell. Both sessions were separated by a period of 48 h. All tests were conducted in the exercise physiology laboratory at the university. Participants were initially briefed. One week was left for voluntary registration. The following week they performed the familiarization and then started the three tests, one each week (Figure 1).

### 2.2. Participants

Fifty male physical activity and sport science students (22.56 ± 3.44 years, 76.61 ± 10.93 kg, 1.79 ± 0.06 m, and a BMI of 23.95 ± 2.73 kg·m^−2^) participated in the present study. Participants were divided into four groups according to their relative strength ratio (RSR), which was obtained based on their 1RM strength/body mass ratio: (1) RSR > 1.25, High RSR (*n* = 11); (2) 1.25 < RSR > 1.05, Medium RSR (*n* = 14); (3) 1.05 < RSR > 0.85, Low RSR (*n* = 14); (4) RSR < 0.85, Very low RSR (*n* = 11). All participants were healthy and without cardiorespiratory, muscular, or metabolic limitations that could limit their performance in the study. Further, no participant used medication, nutritional supplements, or energy drinks for the study duration. Participants were instructed not to consume any type of food over the two hours before the test, with only water being permitted. Further, it was ensured that all participants were aware of how to correctly execute the BP exercise. Participants were encouraged to perform the same training the week before each test. On the day before to the test, participants were encouraged not to exercise. The calculation was performed with an α = 0.05 (5% chance of type I error) and 1–β = 0.85 (power 85%), applying the results of previous studies in which the sample size was the same or smaller. The calculated sample size was 44 subjects. Before the study started, participants were informed about all of the tests to be performed and voluntarily provided written informed consent. The study protocol was approved by the ethical committee at the university, following with the principles stated in the Declaration of Helsinki [21].

### 2.3. Procedures

#### 2.3.1. 1RM Test

The exercise begins in supine position on the bench with hip and knee flexion, placing the feet on the bench. Arms should be held slightly wider than shoulder-width apart. The bar should be lowered slowly and in a controlled fashion towards the chest, slightly above the nipples. The bar should then be held in this position for approximately 1.5 s, and supported over the chest, to eliminate the rebound effect and improve the consistency of repetitions [22]. The order to move into the concentric phase was given verbally by a researcher counting down to 1.5 s from the beginning of the eccentric phase. Participants were encouraged to perform the concentric phase as quickly as possible, without lifting their shoulders or trunk off of the bench and without rebounding. The first test performed by participants constituted a progressive load BP test 1RM. Initially, a warm-up was performed which consisted of 5 min of low-intensity running and 5 min of joint mobility and dynamic stretching exercises. This was followed by one set of 10 repetitions of BP at a fixed load of 10 kg and one set of 5 repetitions of BP at a fixed load of 20 kg. A detailed description of the BP testing protocol has been recently provided elsewhere [11].

#### 2.3.2. Maximum Number of Repetitions (MNR) Test

This test consisted of performing as many repetitions as possible (MNR) against the 70% 1RM, with this load being determined through the MPV obtained from the individual load–velocity relationship. The second test (T2) was performed one week later, on the same day of the week and at the same hours to control for the effects of the circadian rhythm [23]. Just one week after performing T2, the third test was performed, in line with the same schedule, repeating the test (T3). The technical execution of the BP in T2 and T3 was identical to that used in the 1RM test.

#### 2.3.3. Blood Lactate Concentrations

Before the warm-up and 3 min after the MNR test, in both T2 and T3, capillary blood samples were taken from the fleshy part of the finger (5 μL) to determined lactate concentrations.

#### 2.3.4. Measurement Equipment

The test was performed using a multipower, bar-guiding Smith machine (Matrix, Chácara Alvorada, Brazil), inserting 20, 10, 5, 2.5, and 1.25 kg discs (Matrix). In this set-up, both ends of the barbell are fixed allowing only vertical movement of the bar. To estimate the execution velocity of each repetition in different tests, a previously validated opto-electronic instrument was used [24] with a sampling frequency of 500 Hz (Velowin v.1.7.232, Instruments and Sports Technology; Murcia, Spain). The optoelectronic instrument was calibrated according to manufacturer instructions. MPV was calculated automatically using algorithms that are internally generated by the software used for analysis (Velowin v.1.7.232). Lactate measurements were performed with a previously validated and calibrated portable lactate analyzer. (Lactate Pro 2 LT-1710, Arkray Factory Inc., KDK Corporation, Siga, Japan) [25,26].

#### 2.3.5. Examined Variables

In both the 1RM test and in T2 and T3, the MPV of each repetition was measured (bar velocity values during the propulsive phase, defined as the portion of the concentric phase during which bar acceleration is ≥9.81 m·s^−2^). Further, during T2 and T3, the following measurements were made: (1) blood lactate concentrations before and after the test; (2) number of repetitions (MNR 70% Rep); (3) load in kg (70% Load); (4) MPV attained during the best (fastest) repetition of the set (MPVrep Best); (5) MPV attained during the last repetition of the set (MPVrep Last); and (6) loss of MPV (% loss MPV test), defined as (MPVrep Last − MPVrep Best)/MPVrep Best × 100.

### 2.4. Statistical Analysis

Shapiro–Wilk test was used to check the normality of data. Second-order polynomials were used to establish the load–velocity relationship for each subject in the progressive load test to 1RM (T1). In addition, Pearson correlations were used to establish the relationship between the number of repetitions and percentage velocity loss throughout over the course of the set, as well as blood lactate concentrations. A one-way ANOVA was performed to compare differences between participants following the performance of the test at 70% intensity, as a function of strength level. The Bonferroni post-hoc test was used in cases where ANOVA outcomes were significant.

Intra-individual variability between T2 and T3 was examined employing the standard error of measurement (SEM). In addition, Bland–Altman’s systematic bias ± random error and the coefficient of variation (CV), expressed as a percentage of mean outcomes, were used [27]. CV estimations ranged by less than 8.6% [28]. The intra-class correlation coefficient (ICC) was also calculated at a 95% confidence interval (CI). ICC outcomes were classified as follows: excellent reliability (ICC ≥ 0.90), good reliability (0.90 > ICC ≥ 0.70), fair reliability (0.70 > ICC ≥ 0.40), and poor reliability (ICC < 0.40) [29]. A related samples *t*-test was performed to examine the first test (T2) and retest (T3) differences between MNR and MPV for the best and last repetition. All data were expressed as means, standard deviations (SD), 95% CI, and minimum–maximum ranges (Min–Max). The level of significance was set at *p* < 0.05. All statistical tests were performed using the package SPSS version 25.0 (SPSS, Chicago, IL, USA).

## 3. Results

### 3.1. Mean Propulsive Velocity at 1RM

The RSR of the whole group was 1.03 ± 0.23, and the load obtained in the 1RM was 78.72 ± 19.19 kg. Following analysis of MPV at 1RM in the group as a whole, values of 0.16 ± 0.05 m·s^−1^ were documented. Examining this variable within each RSR group, significant differences were found between the MPV High RSR and Very Low RSR (*p* < 0.05) groups, with values being similar in the other groups (0.16–0.18 m·s^−1^).

### 3.2. Maximum Number of Repetitions (MNR) at 70% Intensity

The number of repetitions performed at 70% MPV was 12.38 ± 2.51 overall, with a CV of 20% (Table 1). When the number of repetitions was examined according to the strength group, no significant differences were found (*p* < 0.05), with the number of repetitions being between 11.2 and 13.1. A trend towards more repetitions was found in the groups with higher RSR. Higher RSR led to lower CV values (CV = High RSR = 13%; CV = Medium RSR = 15%, CV = Low RSR = 22%; CV = Very Low RSR = 29%). This suggests that more variability exists in the performance when the individuals have less general strength.

### 3.3. Mean Propulsive Velocity Attained during the Best Repetition at 70% Intensity

No significant differences were found between Medium RSR, Low RSR, and Very Low RSR groups, with highly similar MPV values being obtained in relation to the best repetition performed in the set (0.62 m·s^−1^, 0.63 m·s^−1^, 0.64 m·s^−1^) and low CV values (8–11%). Nonetheless, the group made up of the strongest individuals (High RSR) was significantly different from all other groups (*p* < 0.001), recording a MPV of 0.52 m·s^−1^.

### 3.4. Mean Propulsive Velocity Loss at 70% Intensity

Velocity loss throughout the set was similar in all strength groups (−72.86% ± 7.22–−74.09% ± 7.39), with no significant differences (*p* < 0.05) and acceptable CV (between 10 and 12%) (Table 1). With regards to MPV of the final repetition of each set, no significant differences were observed between groups (*p* < 0.05) (between 0.14 and 0.17 m·s^−1^). CV was approximately 20% within the highest RSR groups (High and Medium), and 30% in the lowest RSR groups (Low and Very Low) (Table 1).

### 3.5. Blood Lactate Concentrations

The overall sample had average concentration of 5.72 mmol·L^−1^, with significant differences between the Medium RSR group and the Very Low RSR group (6.27 ± 1.38 mmol·L^−1^y 4.77 ± 1.37 mmol·L^−1^) (Table 1).

Table 2 presents test-retest variability. No significant differences were found (*p* < 0.05) between T2 and T3 in the number of repetitions achieved at 70% (SEM ≤ 1.17 repetitions), MPVrep Best (SEM ≤ 0.048 m·s^−1^), or MPVrep Last (SEM ≤ 0.035 m·s^−1^), nor were there significant differences between groups (*p* < 0.05). Further, it was observed that within-individual variability of the three aforementioned variables decreased (lower SEM) as the RSR increased. With regards to the number of repetitions performed, the lowest SEM values were recorded for the Medium RSR (SEM ≤ 0.63 repetitions) and High RSR (SEM ≤ 0.67 repetitions) groups, while the highest SEM values were recorded for the Very Low RSR group (SEM ≤ 2.18 repetitions). The reliability of the variables measured at T2 and T3 was good for the number of repetitions at 70% MVP (ICC = 0.777) and MPVrep Best (ICC = 0.716), and there was fair reliability for the MPVrep Last (ICC = 0.400).

It also serves to highlight that, for all 50 participants, CV values for the number of repetitions performed at 70% MPV for MPVrep Best were acceptable (CV = 9.2%, CV = 7.9%, respectively) and for MPVrep Last were high (CV = 22.5%). Considering CV as a function of participant strength level, low values (CV~5%) are observed with the variables of the number of repetitions at 70% and MPVrep Best for the High RSR and the Medium RSR, with acceptable CV values in the Low RSR, and high CV values in Very Low RSR (CV > 10%). The CV values are very high for the MPVrep Last in all strength levels.

**Figure 2:**
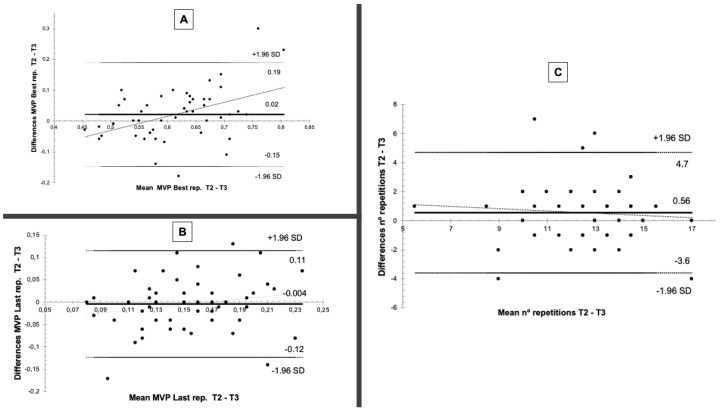
Bland–Altman plots of intra-individual variability (test (T2)-retest (T3)) pertaining to (**A**) mean propulsive velocity attained during the best repetition (MPV Best); (**B**) mean propulsive velocity attained during the last repetition (MPV Last); and (**C**) number of repetitions performed at 70% intensity (MNR).

Within-individual test-retest variability was also examined using Bland–Altman plots. There was a systematic bias for the three analyzed variables in all RSR groups, with the lowest corresponding to High RSR (Table 3). This systematic bias remained low when the overall participant group was examined (Figure 2).

On the other hand, no significant correlations were recorded between the number of repetitions and velocity loss during the MNR, nor between the number of repetitions in the test to failure and post-test blood lactate concentrations (Table 4).

## 4. Discussion

One of the main findings of the present study is that when employing a 70% MNR BP test, following determination of the 1RM in a progressive load test to exhaustion and controlling the test through monitoring of the MPV, intra-individual variability is too high (CV = 13–29%) to support the use of an xRM protocol as a means of managing intensity during BP exercise. This was the case even though the average number of repetitions fell between 11 and 13 (CV = 13–29%), with the relative intensity achieved by each individual being highly variable. This, together with the fact that the MPV achieved during the best repetition, regardless of strength grouping, was within values reported in other studies (0.62–0.64 m·s^−1^), suggests that MPV could be used as a tool to manage training intensity. Further, intra-individual variability outcomes suggest that within this group, this test had high reproducibility and led to consistent outcomes.

When considering data obtained following the performance of the progressive load test until 1RM, obtained velocity values (MPV 1RM: 0.16 ± 0.04 m·s^−1^) were similar to those reported by previous studies [10,18,30]. This is highly relevant not just because it reveals that each exercise is characterized by a specific velocity of about 1RM, but also because it makes clear the need to use velocity as a means to providing a certain degree of security that the absolute load being shifted corresponds to the 1RM of each individual.

Nonetheless, the fact that variation was seen in the present sample in this velocity at 1RM should not be ignored. This was particularly the case in the High RSR group (MPV 1RM: 0.12 ± 0.03 m·s^−1^). We must consider this fact when using the velocity corresponding to the programmed relative intensity (70%), since the velocity related to this intensity may be slightly different. In the same way, the variation found between groups in MPV values of the repetition corresponding to 1RM, with velocities both above and below average values being recorded, should be considered. In contrast to what might have been considered before this research, this could provide an argument against the use of progressive load or other similar tests. This is especially so in individuals with low levels of relative strength (RSR) and lacking in experience with strength training. In this case, the lack of exposure to absolute loads, such as those corresponding to 85% 1RM or higher, could increase levels of muscle coactivation and, in this way, negatively impact test outcomes and, therefore, obtained MPV values.

All this should be considered together with the significant disadvantages of performing a 1RM test (subjecting the individual to high levels of stress and fatigue, great variability, a huge investment of time, need to perform this test for all the exercises selected for a given session, the impossibility of knowing the right moment to perform it, etc.), which make it hard to define training intensity in this way in practice. The velocity values produced during a progressive load test allow for greater accuracy in determining 1RM in individuals.

Outcomes with regards to the MPV values corresponding to 70% exercise intensity (MPVrep Best: 0.62–0.64 m·s^−1^) were highly similar to those obtained in other previous studies [10,31]. These findings would appear to suggest that the measurement of velocity could enable relative intensity to be defined with sufficient accuracy. This would enable achieved MPV to be controlled within individuals when subjected to a determined absolute load, regardless of the relative strength of different individuals. With regards to the variation seen in MNR at a given intensity, values tended to be high (CV: 20%). Where possible, reference values found in the literature for velocity and its relationship with relative intensity should be used [10,31] to prescribe intensity. This could also offer a more realistic approach in practice, as it implies less risk and time investment, while the potential margin of error is narrower and easier to compensate. Such an approach would make it possible to avoid having to perform a progressive load test for every exercise included within a training session. It would also prevent the use of other models which are likely to lead to the use of heavier loads than that programmed for the training session [32,33] or, even, employing xRM to define resistance training intensity. The latter is undesirable given the negative effects associated with this type of maximum effort exercise [34,35,36,37].

Another important issue to emerge is that within certain populations, such as those of individuals with High RSR, velocity corresponding to the programmed intensity may be slightly different, as was the case in the present study (MPVrep Best: 0.52 ± 0.04 m·s^−1^). This outcome was similar to that reported in other studies [30,38]. These differences may be explained by methodological differences in the research design of each study but, also, by the training experience of participants, with athletes tending to train at intentionally low velocities or using relatively high intensities.

Following the performance of an MNR test at 70% intensity, an average number of repetitions of between 11 and 13 was achieved, with no difference between groups. Nonetheless, significant variation existed between groups with regards to MNR (CV: 13–29%), with greater variability in the group corresponding to the lowest RSR values (CV: 29%). This finding is consistent with previously published findings [12,17,18,19]. All of that discussed above leads to the conclusion that the establishment of a similar number of maximum repetitions (xRM) for a specific set of individuals, as has been done often within the literature [39,40,41,42], would not enable intensity to be defined precisely. This would lead some individuals to train at a different intensity to that programmed, with individuals with the least experience and strength being most vulnerable to this issue.

Some observations arising from the collected data can also be made concerning study development. A tendency of participants towards a degree of velocity “self-management” emerged in the first repetitions of a set. This reflected participants dwelling on the aim of performing as many repetitions as possible, despite being informed and motivated to perform each repetition at the highest velocity possible. The aforementioned does not detract from the potential value of employing intensity to determine relative intensity during strength training. Instead, it reflects a margin of error that must be assumed by exercise professionals in light of the more significant drawbacks of alternative options for defining and managing the aforementioned training variable.

Once again, the monitoring of velocity during this test permits a certain degree of security, which would be difficult to achieve without exercising any control over this variable, leading individuals to reach MNR at 70% intensity, in other words, reaching “muscular exhaustion” during the test. This was confirmed by the finding that the MPV achieved during the last repetition of the test corresponding to the same MPV as seen at 1RM (0.16 ± 0.04), in addition to the fact that velocity loss throughout the set did not reveal any significant differences (% MPV loss test: −73.64 ± 7.43; *p* > 0.05; CV: 10–12%). Blood lactate served as an important indicator of metabolic stress (Lactate POST: 5.72 ± 1.50 mmol·L^−1^). Significant differences were seen between groups with Low and Medium RSR (Lactate POST: 6.27 ± 1.38 mmol·L^−1^, y 4.77 ± 1.37 mmol·L^−1^). This may indicate a lesser capacity for cytosolic glycolysis use in the low RSR group.

In this sense, gathered data with regards to intra-individual variability coincided with that reported in other studies [17,20]. This means that little intra-individual variability is seen when the MNR test is performed at two different time-points (T2 and T3). This reflects low SEM values, with SEM being even in individuals with higher levels of strength. Similar outcomes were reported using CV for the number of repetitions at 70% and MPVrep Best. With regards to the overall participant group, values for all variables fell within the established range (between 9.2% and 7.9%). This points to adequate intra-individual variability between the two tests, falling approximately within the range of <8.6% reported by Hopkins et al. (2001) [28]. The range of CVs established by Hopkins et al. (2001) [28] was obtained from measurements taken from an isoinertial test, with 8.6% being the highest value obtained. When the analysis conducted was stratified according to participant strength as a function of RSR, all CVs were observed to be within the established range (for number of repetitions at 70% and MPVrep Best), with the only exceptions in the group made up of the weakest individuals (Very Low RSR) (CVs: 18.5–11.7%). Analysis of intra-individual variability between the two measurements (T2 was T3) was complemented with Bland–Altman plots. Outcomes from this also coincided with SEM and CV outcomes. Thus, after taking measurements from the same individual one week apart, it can be stated that the consistency or stability of measurements taken in the present study is a key factor for ensuring that the variations detected between MNR sessions are not due to systematic bias. Such systematic bias refers to the influence of fatigue, learning effects, or random error due to mechanical or biological variations [43].

Further, good reliability (≥0.70) was reported in terms of the number of repetitions performed at 70% intensity and MPVrep Best. Acceptable reliability (≥0.40) was reported in terms of MPVrep Last. This indicates good consistency of the performed tests and obtained results.

Finally, it serves to highlight that, despite the heterogeneity seen to exist in MNR performed at 70% intensity during BP exercise, velocity loss and blood lactate values were similar between groups. This finding adds further support to the value of controlling velocity to be able to estimate intensity as an independent variable of interventions [10,18] but, also, as a dependent variable to evaluate the potential acute effects of training. Indeed, similar conclusions have been reached by other research studies [44].

Another relevant issue to emerge from the present analysis gives rise to reflections around the fact that, within the training setting, individuals are often encouraged to train at a determined intensity according to xRM (for example, 12). This ensures that each training session is held the same and intended to align the session with the number of maximum repetitions that can be performed by a given individual. The present study suggests that not only could many individuals be engaging in training sessions that differ from the intended purpose, but that this type of methodological approach is difficult to carry out and replicate in practice. It is a challenge for studies to reflect the training performed by presenting the actual number of repetitions performed with the absolute load established for each individual, with this often being determined via progressive load tests to 1RM. This presents a further limitation with regards to identifying the real relative intensity achieved by a given individual.

Thus, the use of xRM (for example, 8RM, 10RM, 15RM, etc.) as a means to control intensity would also be inappropriate and inaccurate, and lead to excessive fatigue when performing the exercises and sets proposed as part of a training session. This leads to the conclusion that controlling execution velocity may offer an appropriate alternative for guaranteeing precise intensity prescription with regard to strength training. This approach also avoids the need to perform a progressive load test to 1RM or another similar test in favor of simply monitoring velocity during training and/or the changes produced throughout.

Our findings apply to individuals characterized by different levels of training experience, although evaluation may be necessary within individuals with experience and/or high levels of strength. This may especially be the case when working at high loads or training at a low velocity, given the variability observed. Nonetheless, to a certain degree, the challenge proposed by the variation seen in some populations can be assumed given the drawbacks of other alternatives.

The limitation is that the present study looked at differences in one relative intensity measure. The results pertain to a large sample and have several practical applications that may be very important and confer significant ecological validity. Other future studies should explore this same issue in others exercises and intensities.

## 5. Conclusions

The use of variables such as the 1RM, estimated using an absolute load value, or a maximum number of repetitions per series (MNR) do not allow an adequate degree of precision to prescribe and control the relative intensity of resistance training. Besides, execution velocity control can offer an adequate alternative to guarantee an accurate prescription of intensity with regard to resistance training. This approach also avoids the need to perform a 1RM progressive load test or other similar tests in favor of simply monitoring velocity during training and/or changes throughout training.

## Figures and Tables

**Table 1:** Data were recorded from the test at a load of 70% 1RM.

	MPV 1 RM (m·s^−1^)	70% Load (kg)	MNR 70% Rep (nº)	% Loss MPV Test	MPV_rep_ Best (m·s^−1^)	MPV_rep_ Last (m·s^−1^)	Lactate PRE (mmol·L^−1^)	Lactate POST (mmol·L^−1^)
(*n* = 50)								
M ± SD	0.16 ± 0.05	54.98 ± 13.12	12.38 ± 2.51	−73.64 ± 7.43	0.60 ± 0.07	0.16 ± 0.04	1.42 ± 0.33	5.72 ± 1.50
95%CI	0.14–0.17	51.25–58.71	11.67–13.09	−71.53–−75.75	0.58–0.62	0.14–0.17	1.32–1.51	5.30–6.1550
Min–Max	0.05–0.30	31–88	5–19	54.21–87.5	0.47–0.76	0.08–0.28	0.90–2.20	3.20–9.80
CV	31%	24%	20%	10%	12%	25%	23%	26%
High RSR (*n* = 11)								
M ± SD	0.12 ± 0.03 †	71.73 ± 10.00 *	12.82 ± 1.72	−72.86 ± 7.22	0.52 ± 0.04 *	0.14 ± 0.03	1.35 ± 0.38	6.14 ± 1.18
95%CI	0.10–0.14	65.02–78.43	11.66–13.97	−68.01–−77.71	0.49–0.55	0.12–0.16	1.10–1.61	5.34–6.93
Min–Max	0.05–0.17	60–88	10–15	61.70–86.44	0.47–0.60	0.08–0.18	0.90–2.20	3.80–7.70
CV	25%	14%	13%	10%	8%	21%	28%	19%
Medium RSR (*n* = 14)								
M ± SD	0.16 ± 0.04	58.50 ± 6.39 £	13.14 ± 2.03	−73.96 ± 7.29	0.62 ± 0.06	0.15 ± 0.03	1.39 ± 0.31	6.27 ± 1.38 ‡
95%CI	0.14–0.18	54.81–62.19	11.97–14.32	−69.75–−78.17	0.58–0.65	0.13–0.17	1.21–1.57	5.47–7.07
Min–Max	0.09–0.25	48–71	8–17	54.21–83.30	0.51–0.73	0.10–0.22	0.90–1.80	4–8.80
CV	25%	11%	15%	10%	10%	20%	22%	22%
Low RSR (*n* = 14)								
M ± SD	0.17 ± 0.06	47.21 ± 7.85	12.21 ± 2.72	−74.09 ± 7.39	0.63 ± 0.05	0.16 ± 0.05	1.42 ± 0.32	5.51 ± 1.63
95%CI	0.13–0.21	42.68–51.74	10.64–13.79	−69.83–−78.35	0.60–0.66	0.13–0.19	1.24–1.60	4.57–6.46
Min–Max	0.08–0.26	38–61	9–19	62.90–87.50	0.54–0.71	0.08–0.27	1.00–2.10	3.70–9.80
CV	35%	17%	22%	10%	8%	31%	23%	30%
Very low RSR (*n* = 11)								
M ± SD	0.18 ± 0.06	43.64 ± 7.65	11.18 ± 3.22	−73.44 ± 8.78	0.64 ± 0.07	0.17 ± 0.05	1.52 ± 0.35	4.77 ± 1.37
95%CI	0.14–0.22	38.50–48.77	9.02–13.34	−67.54–−79.34	0.59–0.69	0.13–0.21	1.29–1.75	3.86–5.69
Min–Max	0.10–0.30	31–53	5–15	61.70–85.25	0.54–0.76	0.09–0.28	1.00–2.10	3.20–8
CV	33%	18%	29%	12%	11%	29%	23%	29%

MPV = mean propulsive velocity; MNR = maximum number of repetitions; RSR = relative strength ratio, defined as 1RM divided by body mass; Rep = repetitions; MPV_rep_ Best = mean propulsive velocity attained during the best repetition; MPV_rep_ Last = mean propulsive velocity attained during the last repetition; Lactate = blood lactate concentration. M = mean ± SD = standard deviation; CI = confidence interval; Min–Max = lowest value–highest value; CV = coefficient of variation. * = significant difference between High RSR and all other groups (*p* < 0.05). **£** = significant difference between Medium RSR and all other groups (*p* < 0.05). **†** = significant difference between High RSR and Very Low RSR (*p* < 0.05). **‡** = significant difference between Medium RSR and Very Low RSR (*p* < 0.05).

**Table 2:** Intrasubject variability in the number of repetitions performed before failure (MNR), MPV_rep_ Best, and MPV_rep_ Last on two different days according to strength (RSR).

	MNR 70% Rep (nº)				MPV_rep_ Best (m·s^−1^)				MPV_rep_ Last (m·s^−1^)			
	T2	T3	SEM	CV	T2	T3	SEM	CV	T2	T3	SEM	CV
(*n* = 50)	12.38 ± 2.51	12.94 ± 2.36	1.165	9.2%	0.60 ± 0.07	0.62 ± 0.11	0.048	7.9%	0.16 ± 0.04	0.15 ± 0.05	0.035	22.5%
High RSR (*n* = 11)	12.82 ± 1.72	13.09 ± 1.7	0.671	5.2%	0.52 ± 0.04	0.53 ± 0.07	0.031	5.8%	0.14 ± 0.03	0.12 ± 0.04	0.033	25%
Medium RSR (*n* = 14)	13.14 ± 2.03	13.64 ± 2.20	0.631	4.7%	0.62 ± 0.06	0.63 ± 0.09	0.034	5.4%	0.15 ± 0.03	0.15 ± 0.06	0.049	32.7%
Low RSR (*n* = 14)	12.21 ± 2.72	12.57 ± 2.03	0.911	7.4%	0.63 ± 0.05	0.66 ± 0.08	0.055	8.5%	0.16 ± 0.05	0.16 ± 0.05	0.028	17.5%
Very Low RSR (*n* = 11)	11.18 ± 3.22	12.36 ± 3.38	2.18	18.5%	0.64 ± 0.07	0.66 ± 0.13	0.076	11.7%	0.17 ± 0.05	0.19 ± 0.05	0.051	28.3%

MPV = mean propulsive velocity; MNR = maximum number of repetitions; RSR = relative strength ratio, defined as 1RM divided by body mass; Rep = repetitions; SEM = standard error of measurement; MPV_rep_ Best = mean propulsive velocity attained during the best repetition; MPV_rep_ Last = mean propulsive velocity attained during the last repetition. Data expressed as mean ± standard deviation. CI = confidence interval.

**Table 3:** Bland–Altman plots of intra-individual variability (test (T2)-retest (T3)) of the number of repetitions performed before failure (MNR), MPV_rep_ Best, and MPV_rep_ Last on two different days according to strength.

	MNR 70% Rep (nº)			MPV_rep_ Best (m·s^−1^)			MPV_rep_ Last (m·s^−1^)		
	Systematic Bias	Random Error	CI (95%)	Systematic Bias	Random Error	CI (95%)	Systematic Bias	Random Error	CI (95%)
High RSR (*n* = 11)	0.27	1.27	2.82 to −2.27	0.010	0.056	0.122 to −0.102	−0.021	0.047	0.073 to −0.115
Medium RSR (*n* = 14)	0.50	1.16	2.82 to −1.82	0.018	0.063	0.143 to −0.107	0.006	0.066	0.125 to −0.138
Low RSR (*n* = 14)	0.36	1.74	3.83 to −3.12	0.030	0.087	0.203 to −0.143	0.006	0.051	0.096 to −0.109
Very Low RSR (*n* = 11)	1.18	3.63	8.44 to −6.07	0.025	0.128	0.282 to −0.231	0.019	0.073	0.166 to −0.127

MPV = mean propulsive velocity; MNR = maximum number of repetitions; RSR = relative strength ratio, defined as 1RM divided by body mass; Rep = repetitions; SEM = standard error of measurement; MPV_rep_ Best = mean propulsive velocity attained during the best repetition; MPV_rep_ Last = mean propulsive velocity attained during the last repetition. Data expressed as mean ± standard deviation. CI = confidence interval.

**Table 4:** Regression and correlation analysis for the four strength groupings.

	Pearson’s Correlation Coefficient	
	*R*	*p*
**All RSR (*n* = 50)**		
Repetitions 70%–% MPV loss test Repetitions 70%–Lactate POST	0.247 0.131	0.364 0.083
**High RSR (*n* = 11)**		
Repetitions 70%–% MPV loss test Repetitions 70%–Lactate POST	0.493 0.141	0.123 0.679
**Medium RSR (*n* = 14)**		
Repetitions 70%–% MPV loss test Repetitions 70%–Lactate POST	0.100 0.144	0.733 0.623
**Low RSR (*n* = 14)**		
Repetitions 70%–% MPV loss test Repetitions 70%–Lactate POST	0.158 −0.307	0.590 0.285
**Very Low RSR (*n* = 11)**		
Repetitions 70%–% MPV loss test Repetitions 70%–Lactate POST	0.365 0.274	0.270 0.414

RSR = relative strength ratio, defined as 1RM divided by body mass; MPV = mean propulsive velocity; Lactate = blood lactate concentration.

## Data Availability

Not applicable.
